# Supplementary material for: Understanding the Radiobiological Mechanisms Induced by 177Lu-DOTATATE in Comparison to External Beam Radiation Therapy
Source: Int J Mol Sci. 2022 Oct 15;23(20):12369. doi: 10.3390/ijms232012369 (PMC9604190; doi:10.3390/ijms232012369)
Supplement: Supplementary file 1 [file ijms-23-12369-s001.zip › ijms-1903538-supplementary.pptx]

## Slide 1
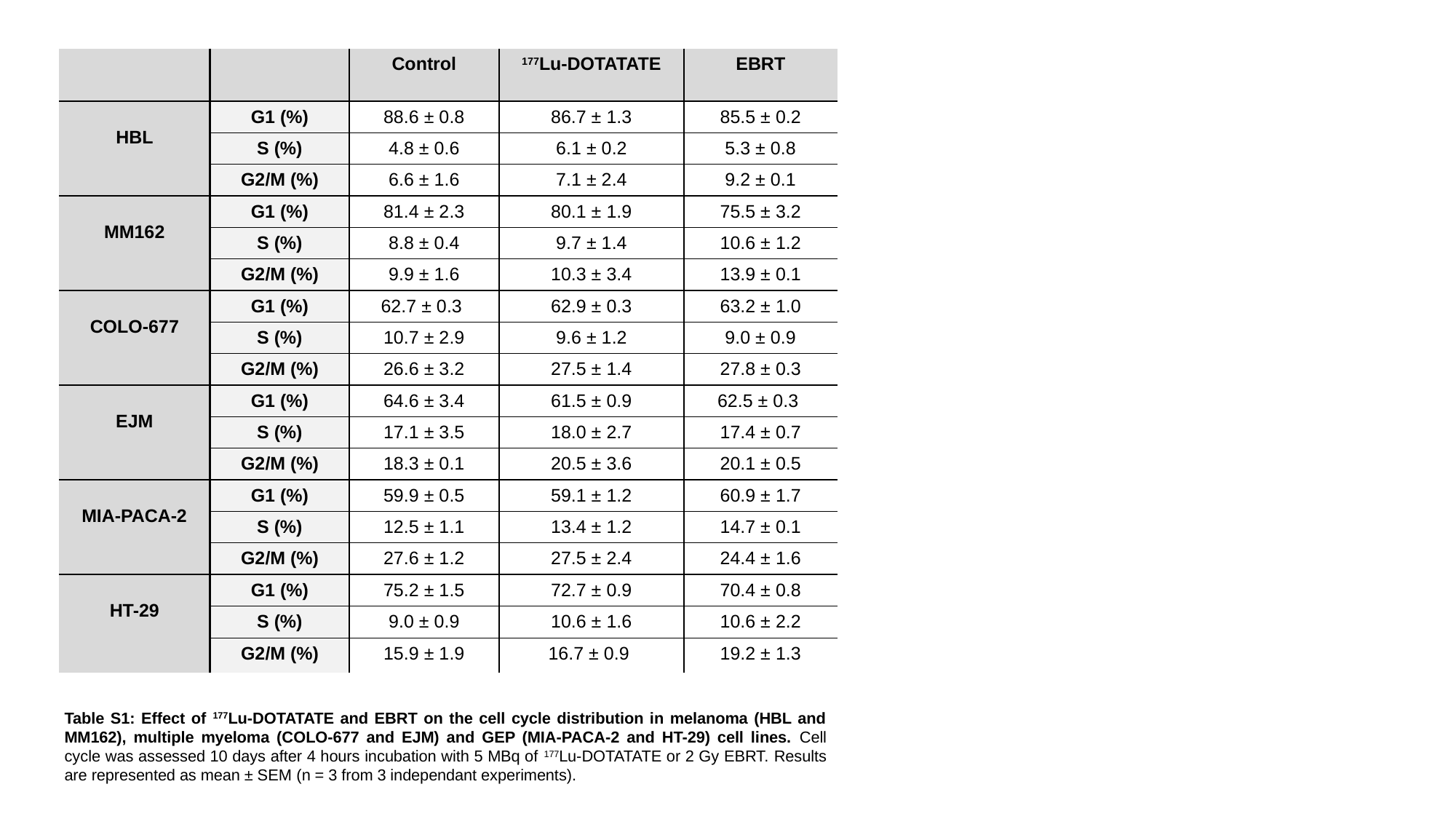

| | | Control | 177Lu-DOTATATE | EBRT |
| --- | --- | --- | --- | --- |
| HBL | G1 (%) | 88.6 ± 0.8 | 86.7 ± 1.3 | 85.5 ± 0.2 |
| | S (%) | 4.8 ± 0.6 | 6.1 ± 0.2 | 5.3 ± 0.8 |
| | G2/M (%) | 6.6 ± 1.6 | 7.1 ± 2.4 | 9.2 ± 0.1 |
| MM162 | G1 (%) | 81.4 ± 2.3 | 80.1 ± 1.9 | 75.5 ± 3.2 |
| | S (%) | 8.8 ± 0.4 | 9.7 ± 1.4 | 10.6 ± 1.2 |
| | G2/M (%) | 9.9 ± 1.6 | 10.3 ± 3.4 | 13.9 ± 0.1 |
| COLO-677 | G1 (%) | 62.7 ± 0.3 | 62.9 ± 0.3 | 63.2 ± 1.0 |
| | S (%) | 10.7 ± 2.9 | 9.6 ± 1.2 | 9.0 ± 0.9 |
| | G2/M (%) | 26.6 ± 3.2 | 27.5 ± 1.4 | 27.8 ± 0.3 |
| EJM | G1 (%) | 64.6 ± 3.4 | 61.5 ± 0.9 | 62.5 ± 0.3 |
| | S (%) | 17.1 ± 3.5 | 18.0 ± 2.7 | 17.4 ± 0.7 |
| | G2/M (%) | 18.3 ± 0.1 | 20.5 ± 3.6 | 20.1 ± 0.5 |
| MIA-PACA-2 | G1 (%) | 59.9 ± 0.5 | 59.1 ± 1.2 | 60.9 ± 1.7 |
| | S (%) | 12.5 ± 1.1 | 13.4 ± 1.2 | 14.7 ± 0.1 |
| | G2/M (%) | 27.6 ± 1.2 | 27.5 ± 2.4 | 24.4 ± 1.6 |
| HT-29 | G1 (%) | 75.2 ± 1.5 | 72.7 ± 0.9 | 70.4 ± 0.8 |
| | S (%) | 9.0 ± 0.9 | 10.6 ± 1.6 | 10.6 ± 2.2 |
| | G2/M (%) | 15.9 ± 1.9 | 16.7 ± 0.9 | 19.2 ± 1.3 |
Table S1: Effect of 177Lu-DOTATATE and EBRT on the cell cycle distribution in melanoma (HBL and MM162), multiple myeloma (COLO-677 and EJM) and GEP (MIA-PACA-2 and HT-29) cell lines. Cell cycle was assessed 10 days after 4 hours incubation with 5 MBq of 177Lu-DOTATATE or 2 Gy EBRT. Results are represented as mean ± SEM (n = 3 from 3 independant experiments).

## Slide 2
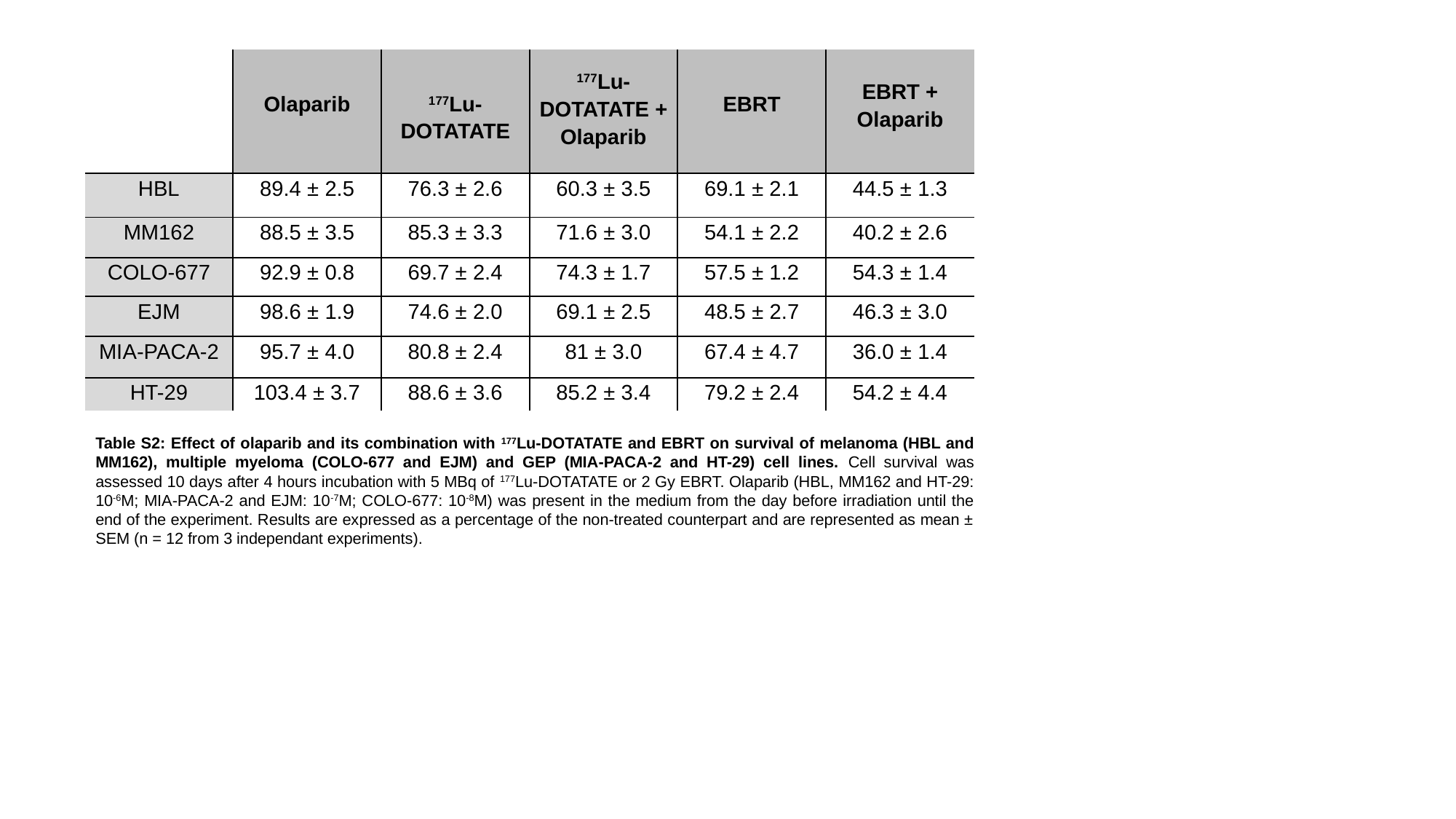

| | Olaparib | 177Lu-DOTATATE | 177Lu-DOTATATE + Olaparib | EBRT | EBRT + Olaparib |
| --- | --- | --- | --- | --- | --- |
| HBL | 89.4 ± 2.5 | 76.3 ± 2.6 | 60.3 ± 3.5 | 69.1 ± 2.1 | 44.5 ± 1.3 |
| MM162 | 88.5 ± 3.5 | 85.3 ± 3.3 | 71.6 ± 3.0 | 54.1 ± 2.2 | 40.2 ± 2.6 |
| COLO-677 | 92.9 ± 0.8 | 69.7 ± 2.4 | 74.3 ± 1.7 | 57.5 ± 1.2 | 54.3 ± 1.4 |
| EJM | 98.6 ± 1.9 | 74.6 ± 2.0 | 69.1 ± 2.5 | 48.5 ± 2.7 | 46.3 ± 3.0 |
| MIA-PACA-2 | 95.7 ± 4.0 | 80.8 ± 2.4 | 81 ± 3.0 | 67.4 ± 4.7 | 36.0 ± 1.4 |
| HT-29 | 103.4 ± 3.7 | 88.6 ± 3.6 | 85.2 ± 3.4 | 79.2 ± 2.4 | 54.2 ± 4.4 |
Table S2: Effect of olaparib and its combination with 177Lu-DOTATATE and EBRT on survival of melanoma (HBL and MM162), multiple myeloma (COLO-677 and EJM) and GEP (MIA-PACA-2 and HT-29) cell lines. Cell survival was assessed 10 days after 4 hours incubation with 5 MBq of 177Lu-DOTATATE or 2 Gy EBRT. Olaparib (HBL, MM162 and HT-29: 10-6M; MIA-PACA-2 and EJM: 10-7M; COLO-677: 10-8M) was present in the medium from the day before irradiation until the end of the experiment. Results are expressed as a percentage of the non-treated counterpart and are represented as mean ± SEM (n = 12 from 3 independant experiments).

## Slide 3
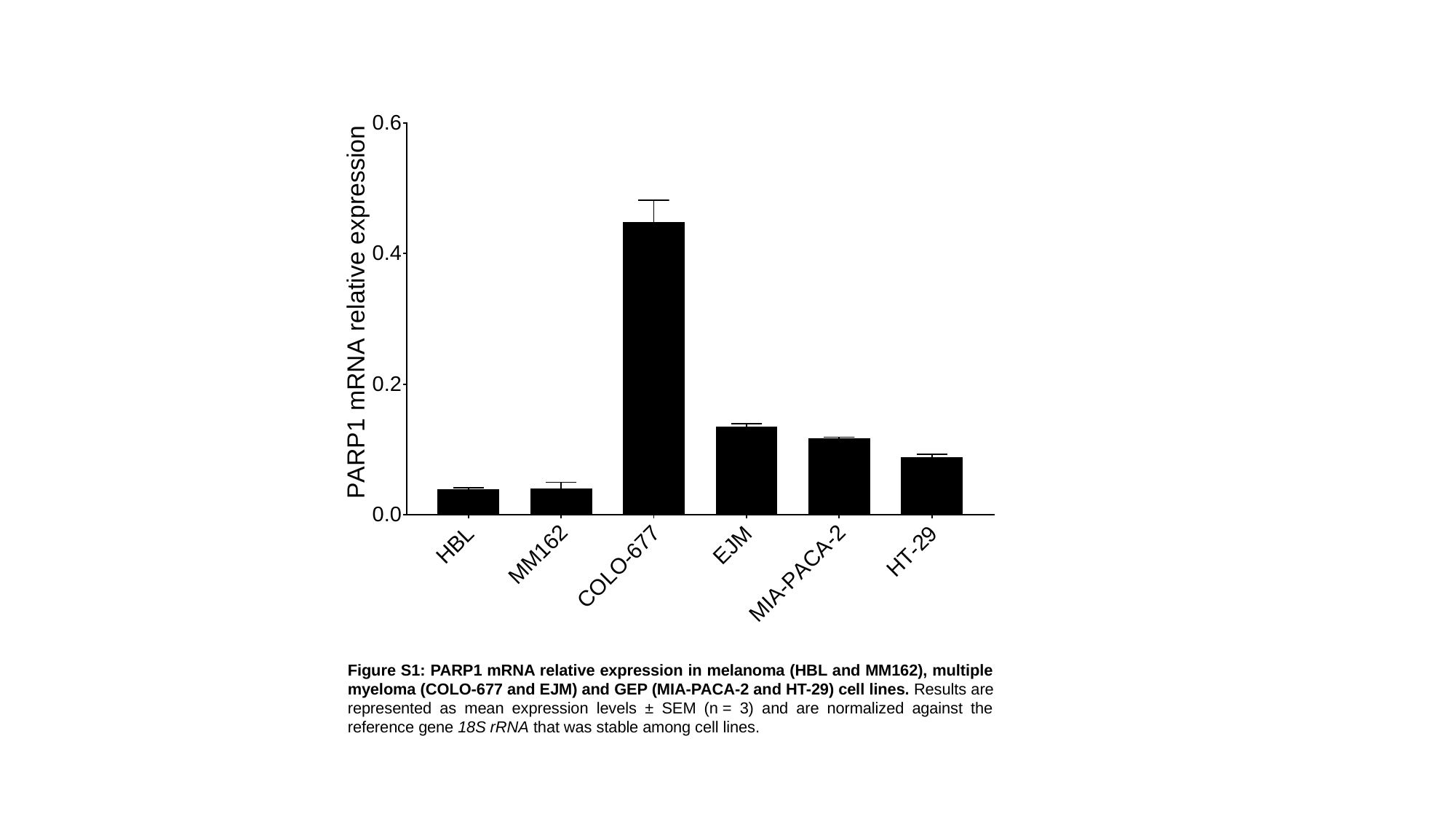

Figure S1: PARP1 mRNA relative expression in melanoma (HBL and MM162), multiple myeloma (COLO-677 and EJM) and GEP (MIA-PACA-2 and HT-29) cell lines. Results are represented as mean expression levels ± SEM (n = 3) and are normalized against the reference gene 18S rRNA that was stable among cell lines.

## Slide 4
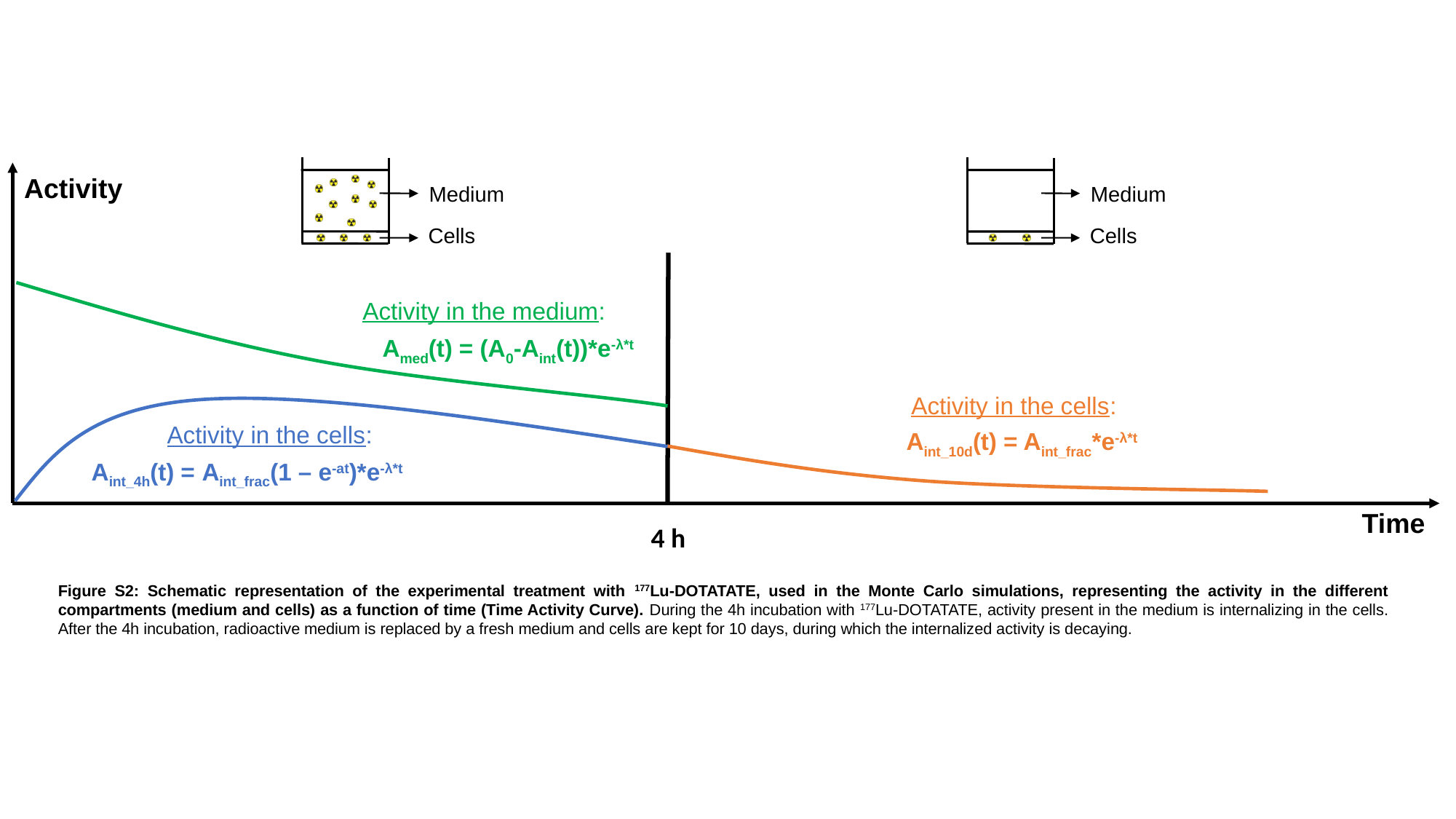

Activity
Time
Medium
Medium
Cells
Cells
4 h
Activity in the medium:
Amed(t) = (A0-Aint(t))*e-λ*t
Activity in the cells:
Activity in the cells:
Aint_10d(t) = Aint_frac*e-λ*t
Aint_4h(t) = Aint_frac(1 – e-at)*e-λ*t
Figure S2: Schematic representation of the experimental treatment with 177Lu-DOTATATE, used in the Monte Carlo simulations, representing the activity in the different compartments (medium and cells) as a function of time (Time Activity Curve). During the 4h incubation with 177Lu-DOTATATE, activity present in the medium is internalizing in the cells. After the 4h incubation, radioactive medium is replaced by a fresh medium and cells are kept for 10 days, during which the internalized activity is decaying.
